# Serum vitamins and microelements levels and their association with children with attention deficit hyperactivity disorder (ADHD)

**DOI:** 10.3389/fmolb.2026.1786113

**Published:** 2026-08-12

**Authors:** Tianzhu Chen, Jiaxiong Yao, Yang Su, Jingya Li, Panli Tan

**Affiliations:** 1 The First Affiliated Hospital of Zhejiang Chinese Medical University (Zhejiang Provincial Hospital of Chinese Medicine), Hangzhou, China; 2 Pharmacy Department, Preparation Center, Nanchang Hongdu Hospital of TCM, Nanchang, China; 3 China Jiliang University, Hangzhou, China

**Keywords:** ADHD, children, microelement, nervous disease, vitamin D

## Abstract

**Background:**

Attention Deficit Hyperactivity Disorder (ADHD) is one of the most common neurodevelopmental disorders in children. The impact of environmental factors on ADHD is very important. Therefore, we analyzed and measured the correlation between the serum vitamin and microelement levels of Chinese children and the incidence rate of ADHD, hoping to provide some guidance for the prevention, diagnosis and treatment of ADHD.

**Methods:**

128 children (69 boys and 59 girls, with an average age of 9.38 ± 2.5 years) were diagnosed with ADHD. In addition, 133 healthy children were recruited as the healthy control group (HC). The levels of serum vitamins and elements were determined by liquid chromatography-mass spectrometry (LC-MS) and inductively coupled plasma mass spectrometry (ICP-MS). Use statistical analysis to compare the differences in the content of two groups of indicators and analyze their correlation with ADHD.

**Results:**

Compared with the healthy control group (HC group), children with ADHD exhibited significantly lower levels of serum vitamin A, vitamin D and vitamin D_3_, while the levels of lead and copper were also significantly elevated. Logistic regression analysis indicated a negative correlation between vitamin D levels and ADHD.

**Conclusion:**

Children with ADHD exhibit significantly lower serum VD levels and higher Pb levels (only in boys), suggesting VD deficiency and sex-specific Pb susceptibility may be key factors associated with ADHD.

## 1 Introduction

Attention deficit hyperactivity disorder (ADHD) is a prevalent neurodevelopmental disorder that primarily affects children and adolescents. Relative to same-age healthy peers, affected individuals exhibit pronounced inattention, shortened attention span, conduct disturbances, hyperactivity, and emotional or behavioral impulsivity, often accompanied by varying degrees of cognitive impairment and learning difficulties (Poddar et al., 2025). The estimated global prevalence of ADHD in children is approximately 3%–10%, with a male to female ratio of 4:1. If not identified and treated during childhood, symptoms may persist into adulthood, substantially impairing patients’ academic, occupational, and daily functioning (Sayal et al., 2018).

Despite extensive investigation, the precise pathogenesis of ADHD remains incompletely understood. Accumulating evidence implicates a multifactorial etiology involving genetic, neurophysiological, neurobiochemical, neuroanatomical, and psychosocial factors, as well as environmental exposures and aberrations in circulating vitamin and microelement levels (Drechsler et al., 2020). Currently, first-line clinical management includes pharmacotherapy, psychological and behavioral interventions, family therapy, and neurofeedback training (Wolraich et al., 2019a). Central nervous system stimulants such as methylphenidate achieve response rates of 75%–80% (Faraone, 2018), whereas atomoxetine, a selective norepinephrine reuptake inhibitor, represents the first non-stimulant approved for ADHD (Mechler et al., 2022).

Several well-established risk factors for ADHD during childhood development include delayed central nervous system maturation, disturbances in neurotransmitter metabolism, adverse living environments, and abnormal serum levels of vitamins and microelements (Dunn et al., 2019; Robberecht et al., 2020; Roy et al., 2021). Vitamins and microelements are indispensable for normal growth and neural development. Vitamins are organic micronutrients obtained from diet; they do not provide energy or serve as structural components but instead mediate intracellular biochemical reactions and regulate systemic metabolism, thereby exerting irreplaceable roles in growth, development, and homeostasis (Tardy et al., 2020). Microelements (e.g., calcium (Ca), iron (Fe), zinc (Zn), lead (Pb), magnesium (Mg), copper (Cu)) constitute less than 0.005%–0.010% of body mass yet possess potent biological activities: they facilitate macroelement transport, serve as essential or auxiliary components of enzymes, hormones, vitamins, and functional proteins, and participate in nucleic acid metabolism and immune regulation (Maggini et al., 2018; Heffernan et al., 2019).

Inadequate intake of essential vitamins/microelements or excessive exposure to toxic elements (e.g., lead) can disrupt metabolic homeostasis, compromise immunity, induce malnutrition, and increase disease susceptibility. Such imbalances may further lead neurological abnormalities, reduced neuronal factors release, blunted responsiveness to psychostimulants, and attention deficits (Wu et al., 2020; Djordjevic et al., 2022). Fluctuations in serum vitamin/microelement levels may contribute to ADHD pathogenesis by affecting neuronal signaling and membrane integrity (Jîtcă et al., 2021). For example, vitamin D (VD) supplementation has been reported to alleviate inattention, hyperactivity, and impulsivity in children with ADHD, an effect thought to be mediated by VD-dopamine interaction in the brain (Hemamy et al., 2021). Similarly, supplementation with Fe and Zn combined with behavioral interventions not only slows disease progression and improve prognosis but alsoboosts immunity (Granero et al., 2021). Collectively, these observations support the notion that abnormalities in circulating vitamin and microelement levels may be causally or consequentially linked to ADHD.

Previous findings on the relationship between specific microelements and ADHD have been inconsistent. Several studies reported no significant difference in serum copper (Cu) levels between ADHD patients and healthy controls (Mahmoud et al., 2011; Yang et al., 2019), whereas others suggests that children susceptible to ADHD exhibit lower Cu concentrations (Kiddie et al., 2010). A recent Mendelian randomization study identified a possible link between copper levels and ADHD, but the association was not significant (Sui et al., 2023). Regarding Mg, deficiency is linked to cognitive impairment, and most investigations have found lower serum Mg levels in ADHD individuals (Hemamy et al., 2021; Hunter et al., 2025), with more severe symptoms correlating with lower Mg concentrations. Nonetheless, a minority of clinical studies have reported comparable or even elevated serum Mg levels in ADHD children relative to controls (Arnold et al., 2005; Irmisch et al., 2011). Additionally, salivary Cu and Zn levels have been associated with higher ADHD prevalence (Robinson et al., 2024), and VD and Mg supplementation may confer benefits for ADHD symptom management (Abhishek et al., 2024). Given these inconsistent findings, the present study further investigated the associations of Cu and Mg with ADHD to contribute additional empirical evidence.

Young children’s inherent high activity levels often cause early ADHD manifestations to be overlooked by families. Nevertheless, as socioeconomic conditions and living standards continue to improve, public awareness of child development has grown, leading to increased attention to ADHD incidence. It is now recommended that interventions begin before school age to prevent adverse effects on children’s academic performance and social development (O’Neill et al., 2017; Wolraich et al., 2019b).

Accordingly, the present study aimed to analyze serum levels of a broad panel of vitamins (VA, VD_2_, VD_3_, VE, VK_1_) and microelements (Cu, Ca, Fe, Mg, Zn, Pb) in children with ADHD compared to healthy controls, and to clarify the relationship between these nutritional/environmental factors and ADHD. Based on existing evidence, we hypothesized that children with ADHD would exhibit significantly lower serum levels of VA, VD, and VE, and significantly higher levels of Pb and Cu, relative to controls. Our findings may provide novel insights for the prevention, clinical diagnosis, and adjunctive treatment of ADHD.

## 2 Materials and methods

### 2.1 Ethical approval of the study protocol

The Research Ethics Committee of the First Affiliated Hospital of Zhejiang Chinese Medical University (Hangzhou, China) approved the study protocol (2022-KL-154–01). Participation in this case-control study was voluntary. Before recruitment, the parents of children suffering from ADHD provided written informed consent.

### 2.2 Exclusion criteria

The exclusion criteria were children: (1) with other neurodevelopmental or neurological disorders (intellectual disorders, autism, epilepsy, neurodegenerative disorders); (2) comorbid psychiatric or medical conditions (anxiety, depression, obesity); (3) with chronic diarrhea, malnutrition, or a history of micronutrient supplementation within the last 3 months; (4) taking medications that may affect the metabolic process of vitamins and microelements such as glucocorticoids, benzodiazepines and other drugs that may affect liver and kidney function.

### 2.3 Enrollment

This study enrolled children who visited the Department of Pediatrics at Zhejiang Provincial Hospital of Chinese Medicine for the first time between January 2021 and December 2024. The diagnosis of attention deficit hyperactivity disorder (ADHD) was established according to the Diagnostic and Statistical Manual of Mental Disorders, Fifth Edition (DSM-5) criteria (Battle, 2013). A pediatric specialist conducted face-to-face interviews with the parents using the Diagnostic Interview Schedule for Children Version IV (DISC-IV) to confirm the diagnosis. Inclusion criteria required the endorsement of at least six symptoms of inattention and/or hyperactivity-impulsivity, with onset before the age of 12 years, and evidence of cross-situational functional impairment. Additionally, all enrolled children had not used any ADHD medication.

### 2.4 Study cohort

Ultimately, 128 children (69 boys and 59 girls) met the criteria for ADHD, with an average age of 9.38 ± 2.5 years. The healthy control group (HC) consisted of 133 children (57 boys and 76 girls) with an average age of 9.42 ± 2.4 years. Children in the HC group were recruited from the Department of Pediatrics of Zhejiang Provincial Hospital of Chinese Medicine and conducted adolescent health check-up during the same period. They did not have neurodevelopmental disorders or other symptoms of disorders related to vitamins or microelements homeostasis.

### 2.5 Laboratory measurements

After fasting overnight, venipuncture was performed, and peripheral blood samples (3 mL) of each child were collected, and then serum was separated by centrifugation (within 6 h). Serum samples were stored at-20 °C. The experiments were conducted by Hangzhou Health Bank Medical (Hangzhou, China). Using a 50 μL sample volume, the levels of serum vitamins (VA, VD, VD_2_, VD_3_, VE, and VK_1_) were determined using liquid chromatography-mass spectrometry (LC-MS), while the levels of microelements (Cu, Ca, Fe, Mg, Zn, and Pb) were determined using inductively coupled plasma mass spectrometry (ICP-MS). The detection limits and reference intervals for serum vitamin and microelement levels are detailed in Tables 1, 2.

**TABLE 1:** The Therapeutic range for serum vitamins levels.

Variable	Therapeutic range	Detection limits (ng/mL)
Serum VA	Deficiency: <0.20 μg/mL Marginal deficiency: 0.20–0.30 μg/mL Normal: 0.30–0.70 μg/mL	5–100000
Serum VD_2_	/	3–1734.2
Serum VD_3_	/	3–9,961
Serum VD	Seriously deficiency: ≤5.00 ng/mL Deficiency: 5.01–15.00 ng/mL Insufficiency: 15.01–20.00 ng/mL Normal: 20.01–100.00 ng/mL Excess: >100.00 ng/mL	/
Serum VE	Normal: 3.80–18.40 μg/mL	150–199480
Serum VK1	Normal: 0.20–2.50 ng/mL	0.15–208

**TABLE 2:** The reference standards for serum microelements levels.

Variable	Reference intervals	Detection limits (ng/mL)
Serum Cu	Normal: 9.42–27.06 μmol/L	4.945–20000
Serum Ca	Normal: 1.35–2.25 mmol/L	36.827–20000
Serum Fe	Normal: 6.50–11.29 mmol/L	46.553–200000
Serum Mg	Normal: 1.35–2.07 mmol/L	4.021–20000
Serum Zn	Normal: 46.27–128.39 μmol/L	8.560–20000
Serum Pb	Normal: 0.00–100.00 μg/L	0.082–200

### 2.6 Statistical analysis

SPSS v26.0 (IBM, Armonk, NY, United States) was used to analyze the data. The measurement data were expressed as mean ± standard deviation, and comparisons between groups were performed using the t-test. The count data were expressed as rates (%), and comparisons between groups were performed using the chi-squared (χ^2^) test. Correlation analysis was conducted using binary logistic regression analysis. *P* value of <0.05 was considered statistically significant.

## 3 Results

### 3.1 Serum levels of vitamins and microelements in the HC group and ADHD group

**FIGURE 1:**
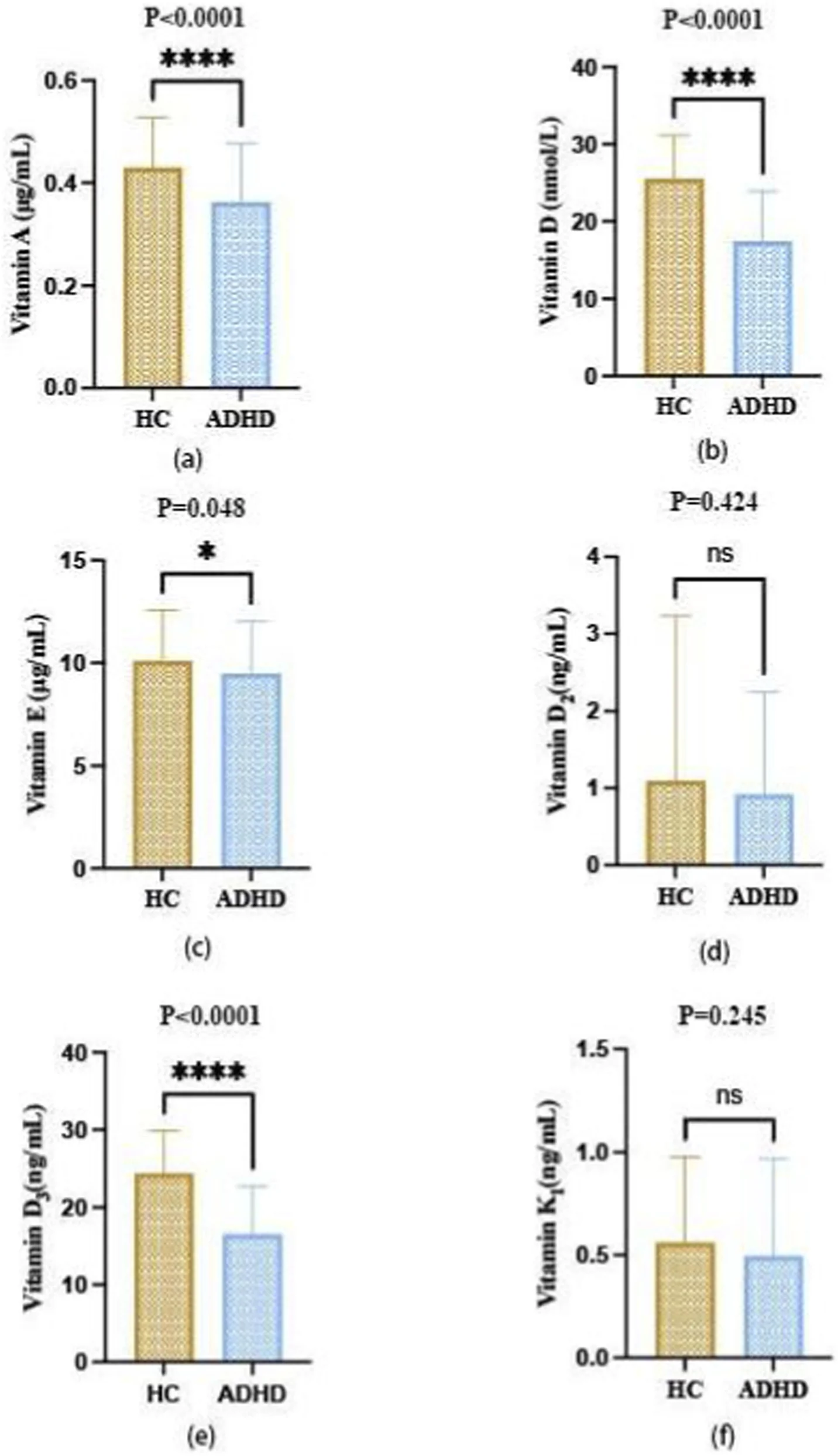
Serum vitamin levels in HC and ADHD groups. HC, Health control (n = 133); ADHD, Attention Deficit Hyperactivity Disorder (n = 128) **(a–f)** Statistical analysis results of vitamin A, D, D2, D3, E, and Kl levels in each group (mean ± SD). *P < 0.05, ****P < 0.0001.

**FIGURE 2:**
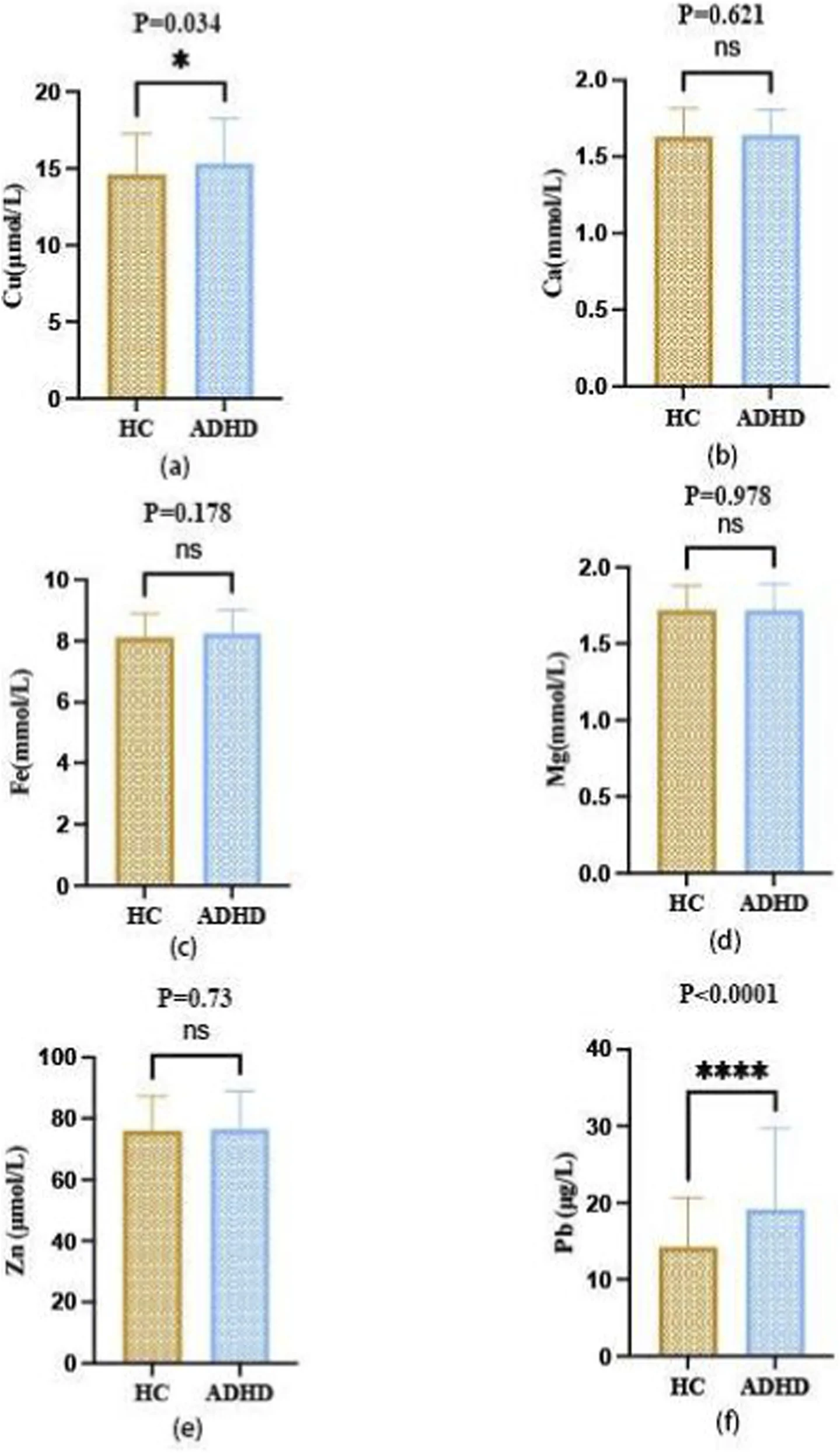
Serum microelements levels in HC and ADHD groups. HC, Health control (n = 133); ADHD, Attention Deficit Hyperactivity Disorder (n = 128). **(a–f)** Statistical analysis results of Cu, Ca, Fe, Mg, Zn, and Pb levels in each group (mean ± SD). *P < 0.05, ****P < 0.0001.

Serum levels of vitamins and microelements in the two groups are presented in Figures 1, 2, and Table 3. Compared with the HC group, children with ADHD exhibited significantly lower serum concentrations of VA, VD, VD_3_ and VE. Meanwhile, serum Pb and Cu levels were significantly higher in the ADHD than in the HC group. However, no significant differences were observed in Fe, Zn, Ca and Mg levels between the two groups. Given that both micronutrient levels and ADHD prevalence exhibit sex differences, this study further stratified the analyses by sex and presented the relevant results separately for boys and girls (Tables 4, 5). After stratifying by sex, the results showed that levels of vitamin A, vitamin D3, and vitamin D in ADHD patients were significantly lower than those in same-sex healthy controls in both boys and girls. Additionally, a significantly higher blood lead level in the ADHD group compared with the control group was observed only in boys. Based on the reference intervals established by the laboratory, we classified VA, VE, VD, VK_1_, Zn, Cu, Fe, Ca, and Mg into three groups: Deficiency, Normal, and Excess, and calculated the corresponding percentages. The remaining parameters were all within the normal range and were therefore excluded from the statistical analysis (Table 6). Markedly higher deficiency rates of VA, VD, and VK1 were observed in the ADHD group.

**TABLE 3:** Serum levels of vitamins and microelements in the HC group and ADHD group.

Variable	HC	ADHD	χ^2^/t	*P*	Cohen’s d (95% CI)
Number of participant	133	128			
Sex			3.189	0.074	
Male	57 (42.86%)	69 (53.91%)			
Female	76 (57.14%)	59 (46.09%)			
Age (years)	9.42 ± 2.4	9.38 ± 2.5	−0.153	0.878	−0.02 (-0.26,0.22)
Serum VA (μg/mL)	0.43 ± 0.10	0.36 ± 0.11	−5.1	<0.0001****	−0.63 (-0.88,-0.38)
Serum VD_2_ (ng/mL)	1.10 ± 2.14	0.92 ± 1.33	−0.8	0.424	−0.1 (-0.34,0.14)
Serum VD_3_ (ng/mL)	24.40 ± 5.43	16.56 ± 6.18	−10.905	<0.0001****	−1.35 (-1.62,-1.08)
Serum VE (μg/mL)	10.10 ± 2.47	9.48 ± 2.54	−1.984	0.048*	−0.25 (-0.49,0)
Serum VD (ng/mL)	25.50 ± 5.61	17.48 ± 6.41	−10.766	<0.0001****	−1.33 (-1.6,-1.06)
Serum VK_1_ (ng/mL)	0.56 ± 0.42	0.50 ± 0.47	−1.164	0.245	−0.14 (-0.39,0.1)
Serum Pb (μg/L)	14.19 ± 6.50	19.15 ± 10.55	4.548	<0.0001****	0.56 (0.32,0.81)
Serum Zn (μmol/L)	75.93 ± 11.25	76.44 ± 12.36	0.346	0.73	0.04 (-0.2,0.29)
Serum Cu (μmol/L)	14.60 ± 2.66	15.33 ± 2.9	2.126	0.034*	0.26 (0.02,0.51)
Serum Fe (mmol/L)	8.12 ± 0.78	8.25 ± 0.77	1.351	0.178	0.17 (-0.08,0.41)
Serum Ca (mmol/L)	1.63 ± 0.18	1.64 ± 0.16	0.495	0.621	0.06 (-0.18,0.3)
Serum Mg (mmol/L)	1.72 ± 0.16	1.72 ± 0.17	−0.027	0.978	0 (-0.25,0.24)

*:*P* < 0.05, **:*P* < 0.01, ***:*P* < 0.001, ****:*P* < 0.0001 compared with the HC, group, n (%), median.

**TABLE 4:** Serum levels of vitamins and microelements in the HC group and ADHD group (boys).

Variable	HC (n = 57)	ADHD (n = 69)	χ^2^/t	*P*	Cohen’s d (95% CI)
Age (years)	10.04 ± 2.88	9.94 ± 2.74	0.185	0.853	0.04 (−0.31, 0.39)
Serum VA (μg/mL)	0.43 ± 0.11	0.37 ± 0.12	3.16	0.002**	0.57 (0.21, 0.93)
Serum VD_2_ (ng/mL)	1.12 ± 2.43	1.04 ± 1.57	0.219	0.827	0.04 (−0.31, 0.39)
Serum VD_3_ (ng/mL)	24.91 ± 6.59	16.54 ± 6.32	7.254	<0.001***	1.30 (0.90, 1.70)
Serum VE (μg/mL)	9.65 ± 2.33	9.29 ± 2.25	0.873	0.384	0.16 (−0.19, 0.51)
Serum VD (ng/mL)	26.03 ± 6.31	17.58 ± 6.57	7.308	<0.001***	1.31 (0.91, 1.71)
Serum VK_1_ (ng/mL)	0.60 ± 0.55	0.49 ± 0.54	1.124	0.263	0.20 (−0.15, 0.55)
Serum Pb (μg/L)	14.19 ± 6.44	21.96 ± 11.02	−4.698	<0.001***	−0.84 (−1.21, −0.47)
Serum Zn (μmol/L)	77.15 ± 10.67	78.89 ± 12.07	−0.852	0.396	−0.15 (−0.50, 0.20)
Serum Cu (μmol/L)	15.21 ± 2.99	15.66 ± 2.92	−0.86	0.392	−0.15 (−0.50, 0.20)
Serum Fe (mmol/L)	8.31 ± 0.83	8.47 ± 0.79	−1.147	0.254	−0.21 (−0.56, 0.14)
Serum Ca (mmol/L)	1.61 ± 0.21	1.62 ± 0.16	−0.269	0.789	−0.05 (−0.40, 0.30)
Serum Mg (mmol/L)	1.73 ± 0.15	1.71 ± 0.17	0.805	0.422	0.14 (−0.21, 0.49)

*:*P* < 0.05, **:*P* < 0.01, ***:*P* < 0.001 compared with the HC, group, n (%), median.

**TABLE 5:** Serum levels of vitamins and microelements in the HC group and ADHD group (girls).

Variable	HC (n = 77)	ADHD (n = 58)	χ^2^/t	*P*	Cohen’s d (95% CI)
Age (years)	8.94 ± 1.85	8.74 ± 1.90	0.597	0.552	0.11 (−0.23, 0.45)
Serum VA (μg/mL)	0.42 ± 0.09	0.36 ± 0.11	3.929	<0.001***	0.70 (0.34, 1.06)
Serum VD_2_ (ng/mL)	1.08 ± 1.89	0.78 ± 0.98	1.103	0.272	0.20 (−0.14, 0.54)
Serum VD_3_ (ng/mL)	23.92 ± 4.45	16.60 ± 6.11	8.06	<0.001***	1.44 (1.04, 1.84)
Serum VE (μg/mL)	10.46 ± 2.53	9.66 ± 2.84	1.722	0.087	0.31 (−0.03, 0.65)
Serum VD (ng/mL)	24.99 ± 5.09	17.37 ± 6.33	7.758	<0.001***	1.38 (0.99, 1.78)
Serum VK_1_ (ng/mL)	0.53 ± 0.28	0.50 ± 0.40	0.46	0.647	0.08 (−0.26, 0.42)
Serum Pb (μg/L)	14.30 ± 6.61	15.76 ± 9.03	−1.085	0.28	−0.19 (−0.53, 0.15)
Serum Zn (μmol/L)	75.18 ± 11.66	73.34 ± 12.15	0.894	0.373	0.16 (−0.18, 0.50)
Serum Cu (μmol/L)	14.73 ± 2.80	14.71 ± 2.25	0.089	0.93	0.01 (−0.33, 0.35)
Serum Fe (mmol/L)	7.98 ± 0.71	7.97 ± 0.67	0.117	0.908	0.02 (−0.32, 0.36)
Serum Ca (mmol/L)	1.64 ± 0.17	1.66 ± 0.16	−0.613	0.541	−0.11 (−0.45, 0.23)
Serum Mg (mmol/L)	1.71 ± 0.16	1.73 ± 0.17	−0.739	0.461	−0.13 (−0.47, 0.21)

*:*P* < 0.05, **:*P* < 0.01, ***:*P* < 0.001 compared with the HC, group, n (%), median.

**TABLE 6:** The categorical distributions of serum vitamins.

Groups		Deficiency	Normal	Excess
Vitamin A	HC	9 (6.8%)	124 (93.2%)	0
	ADHD	44 (34.4%)	84 (65.6%)	0
Vitamin E	HC	0	133 (100%)	0
	ADHD	1 (0.8%)	127 (99.2%)	0
Vitamin D	HC	0	133 (100%)	0
	ADHD	95 (74.2%)	33 (25.8%)	0
Vitamin K_1_	HC	0	133 (100%)	0
	ADHD	33 (25.8%)	94 (73.4%)	1 (0.8%)
Zn	HC	1 (0.8%)	132 (99.2%)	0
	ADHD	1 (0.8%)	127 (99.2%)	0
Cu	HC	0	133 (100%)	0
	ADHD	0	127 (99.2%)	1 (0.8%)
Fe	HC	2 (1.5%)	131 (98.5%%)	0
	ADHD	1 (0.8%)	127 (99.2%)	0
Ca	HC	2 (1.5%)	131 (98.5%%)	0
	ADHD	0	128 (100%)	0
Mg	HC	0	133 (100%)	0
	ADHD	1 (0.8%)	127 (99.2%)	0

### 3.2 Differences of vitamins and microelements between ADHD patients of different genders

For the gender analysis, we assigned scores to males and females for ease of analysis, where males = 1 and females = 2. We analyzed the serum vitamins and microelements of ADHD patients at the level of gender. We found that the serum levels of Pb, Zn, and Cu in patients of different genders were significantly different, and the levels in male patients were significantly higher than those in female patients (Table 7).

**TABLE 7:** Difference of various indicators between ADHD patients of different gender.

Groups	Male 69 (53.9%)	Female 59 (46.1%)	*P* value	F value
VA (μg/mL)	0.37 ± 0.12	0.35 ± 0.11	0.441	1.297
VD_2_ (ng/mL)	1.04 ± 1.57	0.78 ± 0.97	0.258	2.627
VD_3_ (ng/mL)	16.54 ± 6.32	16.58 ± 6.06	0.970	1.086
VE (μg/mL)	9.29 ± 2.25	9.70 ± 2.84	0.363	1.591
VD (nmol/L)	17.58 ± 6.57	17.36 ± 6.28	0.844	1.095
VK_1_ (ng/mL)	0.49 ± 0.54	0.50 ± 0.39	0.931	1.887
Pb (μg/L)	21.96 ± 11.02	15.86 ± 8.99	<0.001***	1.504
Zn (μmol/L)	78.89 ± 12.07	73.57 ± 12.17	0.015*	1.018
Cu (μmol/L)	15.66 ± 2.92	14.95 ± 2.86	0.165	1.039
Fe (mmol/L)	8.47 ± 0.79	7.98 ± 0.67	<0.001***	1.404
Ca (mmol/L)	1.62 ± 0.16	1.66 ± 0.16	0.168	1.022
Mg (mmol/L)	1.71 ± 0.17	1.73 ± 0.17	0.594	1.073

*P < 0.05; ***P < 0.001.

### 3.3 Binary logistic regression analysis of serum vitamins and microelements in ADHD

**FIGURE 3:**
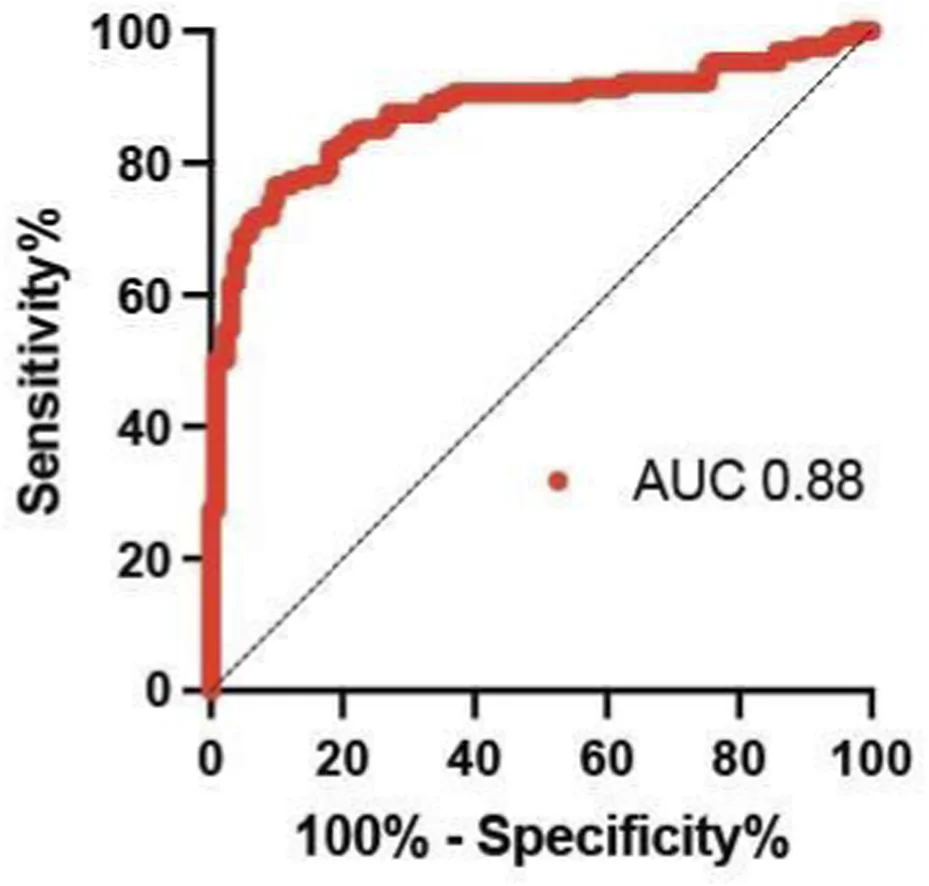
ROC curve analysis based on the combination of serum vitamins and microelements. This curve illustrates the diagnostic performance of the combined panel (Vitamin A, E, D, and trace elements Cu, Pb) in distinguishing the ADHD group from the HC group. The area under the curve (AUC) was 0.88, indicating that this combined model effectively differentiates between the two groups and suggests a synergistic effect of these nutritional elements in the context of this disease.

We conducted linear regression analysis on the vitamins and microelements associated with the measured outcomes and identified a linear relationship between VD (VIF = 16.57) and VD_3_ (VIF = 16.437). Given that VD_3_ is a subtype of VD, VD_3_ was excluded from further analysis, and binary logistic regression was subsequently performed to identify independent influencing factors. The model yielded an *R* ^2^ value is 0.498, AUC = 0.88. *P* < 0.05 was considered statistically significant. The results demonstrated that serum levels of VA (OR = 0.129), VE (OR = 0.985), and VD (OR = 0.774) were inversely associated with ADHD. In contrast, elevated levels of Pb (OR = 1.049) and Cu (OR = 1.040) were associated with ADHD. Notably, VD levels exhibited a highly significant negative association with ADHD (P < 0.001) (Table 8; Figure 3).

**TABLE 8:** Binary logistic regression analysis of the associations between vitamins (A, E, D), microelements (Cu, Pb) and ADHD.

Predictor	B (SE)	Wald χ^2^	P-value	Adjusted OR	95% CI
Demographic factors					
Age (per 1-year increase)	−0.13 (0.08)	2.585	0.108	0.88	(0.75,1.03)
Sex (Ref: Male)	0.4 (0.35)	1.336	0.248	1.498	(0.76,2.97)
Fat-soluble vitamins					
Vitamin A	−2.05 (1.63)	1.587	0.208	0.129	(0.01,3.12)
Vitamin D	−0.26 (0.04)	45.047	<0.001****	0.774	(0.72,0.83)
Vitamin E	−0.02 (0.07)	0.053	0.818	0.985	(0.86,1.12)
Essential metals & minerals					
Lead (Pb)	0.05 (0.03)	3.751	0.053	1.049	(1,1.1)
Copper (Cu)	0.04 (0.07)	0.369	0.544	1.04	(0.92,1.18)

Model Summary: N = 261. The full model was statistically significant: χ2 (14) = 121.94, p < 0.001. Hosmer-Lemeshow goodness-of-fit test: χ2 (8) = 55.58, p < 0.001. Nagelkerke R^2^ = 0.5.

## 4 Discussion

This study demonstrated that the serum concentrations of VA, VD_3_, VE, and VD were significantly lower in children with ADHD than in HC. The deficiency rates of VA, VD, and VK_1_ were also markedly higher in the ADHD group (34.4%, 74.2%, and 25.8%, respectively) compared with HC group (6.8%, 0%, and 0%, respectively), as shown in Table 6. In contrast, serum levels of Cu and Pb were elevated in the ADHD group and showed significant associations with ADHD. These findings suggest that abnormal serum levels of vitamin and microelement are linked to the occurrence and progression of ADHD. After adjusting for collinearity, binary logistic regression analysis further confirmed a strong negative association between VD levels and ADHD.

Existing studies generally indicate that ADHD patients often exhibit deficiencies in Mg, Zn, Fe, and VD, and supplementation with Fe and Zn has been shown to confer benefits in the management of ADHD (Lange et al., 2023). However, in the present study, we did not observe significant differences in serum Mg, Zn, or Fe levels between the ADHD and HC groups (Table 3), and the deficiency rates of these microelements were low in both groups (≤1.5%, Table 6). These discrepancies may be attributable to differences in dietary patterns, geographic location, or socioeconomic status across study populations. Research regarding the roles of VE and VD subtypes in ADHD remains limited.

VD is a neuroactive steroid involved in neuronal differentiation and nervous system development. It can promote the growth and differentiation of nerve cells and regulate immune function (Charoenngam and Holick, 2020; Pignolo et al., 2022). VD receptors and metabolic enzymes are primarily localized in the neurons of the substantia nigra, hippocampus, hypothalamus and prefrontal cortex--brain regions that exhibit abnormalities in patients with ADHD (Tsiaras and Weinstock, 2011; Fairchild, 2012; Tomasi and Volkow, 2014). Other studies have reported that VD can promote the release of neurotrophic factors, participate in the maintenance of neuronal Ca homeostasis, reduce Ca-induced excitotoxic damage, and help preserve normal autonomic nerve function. Collectively, these effects contribute to improved neural stability and promote neural repair. Preservation of dopaminergic neuron function is critical for normal nervous system activity and mental health (Pertile et al., 2023). VD deficiency may contribute to increased mood swings, impaired attention and poor response inhibition in children (Talib et al., 2024). Notably, VD levels have been found to be significantly lower in patients with ADHD comorbid with cognitive dissonance syndrome than in both ADHD patient without comorbidity and HC group (Akman and Sarıgedik, 2025). However, the precise mechanism by which VD deficiency contributes to the pathogenesis ADHD remains unclear. Studies suggest that this association may be related to alterations and impairments in various neurotransmitters, within the brain induced by insufficient VD (Saedisomeolia et al., 2018). VD is mainly derived from dietary and sunlight exposure. VD_3_ is synthesized in human skin upon UVB radiation, whereas VD_2_ can only be obtain through dietary sources (Wilson et al., 2017). These observations highlight that the impacts of diet and sunlight exposure on VD_2_ and VD_3_ status should not be overlooked. Studies have shown that VD_2_ and VD_3_ are metabolically interrelated and share common regulatory pathways (Hammami et al., 2019). Due to the retrospective design of the present study, statistical data regarding sunlight exposure and dietary intake were not available. Further assessments of these potential confounding factors are warranted to clarify their contributions to the observed differences in VD subtypes.

VA is a fat soluble micronutrient that is converted to retinoic acid at or near its site of action in the body, where it functions as a transcriptional regulator (Olson and Mello, 2010). Research regarding the role of VA in ADHD remains limited; however, existing studies have demonstrated that VA levels are lower in patients with ADHD and comorbid conduct disorder (CTD) compared with HC (Wang et al., 2024; Li et al., 2025). These findings are consistent with the results of the present study. Moreover, the high deficiency rate of VA in the ADHD group (34.4%, Table 6) suggests that VA insufficiency may be a common comorbidity in ADHD, potentially contributing to visual attention deficits given VA’s established role in retinal function and visual processing.

VE is a fat soluble vitamin comprising eight natural isomers: α-, β-, δ-,and γ-tocopherols, as well as α-, β-, δ-, and γ-tocotrienols (Abraham et al., 2019). It is commonly associated with multiple health-promoting properties, including antioxidant, anti-inflammatory, cholesterol lowering, and neurotrophic effects (Regner-Nelke et al., 2021). VE exerts neuroprotective effects in the central nervous system and is involved in neurogenesis, neuronal differentiation, hippocampal synaptic function, and cellular signaling pathways (La Torre et al., 2021). Increased oxidative stress and subsequent mitochondrial dysfunction have been observed in individuals with ADHD. Combined supplementation with coenzyme Q10 panthenol, VE, and VB has been shown to alleviate clinical symptoms by improving brain mitochondrial function and ATP production (Cucinotta et al., 2022). We therefore hypothesize that children with ADHD may require higher serum levels of antioxidants such as VE to counteract elevated oxidative stress in the body. Although VE levels were significantly lower in the ADHD group in unadjusted analysis (P = 0.048, Table 3), this difference did not remain significant in the multivariate regression model (Table 8), suggesting that the association between VE and ADHD may be partially confounded by other factors such as VD levels. Future studies investigating the therapeutic effects of VE supplementation on ADHD are thus warranted.

Cu is involved in catecholamine metabolism via β-hydroxylase and monoamine oxidase (MAO). Higher Cu concentration leads to decreased dopamine levels in rats (Yu et al., 2008). Therefore, we hypothesize that increased Cu intake may lead to a decreased dopamine levels, thereby contributing to the development of ADHD. However, in the present study, although serum Cu levels were significantly higher in the ADHD group in unadjusted analysis (P = 0.034, Table 3), this association did not persist after adjusting for other covariates in the regression model (OR = 1.040, P = 0.544, Table 8). Moreover, only one child in the ADHD group (0.8%) exhibited Cu excess (Table 6). These findings suggest that Cu may not be an independent risk factor for ADHD in our study population, and the observed unadjusted difference may be attributable to confounding by other variables. The precise mechanism underlying the association between Cu and ADHD remains unclear, warranting further investigation.

Pb is a global environmental pollutant that can impair brain development and subsequent neurobehavioral outcomes even at low exposure levels (Dórea, 2019). Pb exposure can disrupt dopaminergic signaling pathways, and accumulating evidence has demonstrated that Pb-induced neurotransmitter imbalances may synergistically contribute to the emergence of ADHD-like behavioral phenotypes (Son et al., 2025). These findings suggest that controlling Pb exposure may serve as an effective strategy for the prevention of ADHD. In our study, Pb levels were significantly elevated in the ADHD group (P < 0.0001, Table 3) and showed a borderline positive association with ADHD in the multivariate regression model (OR = 1.049, 95% CI: 1.00–1.10, P = 0.053, Table 8). Notably, the sex-stratified analysis revealed that elevated Pb levels were associated with ADHD only in boys (P < 0.001, Table 4) but not in girls (P = 0.28, Table 5). This sex-specific finding is clinically important and suggests that boys may be more susceptible to Pb-induced neurodevelopmental impairments. Routine Pb screening, particularly in boys with ADHD symptoms, may be warranted in clinical practice.

Our study has several strengths, including the comprehensive panel of vitamins and microelements measured, the inclusion of sex-stratified analyses, and the use of rigorous laboratory methods (LC-MS and ICP-MS). The finding of a high prevalence of VK_1_ deficiency in ADHD (25.8%) is novel and deserves further exploration in future studies.

Our research summarizes the associations between vitamins, microelements, and ADHD. Regular monitoring of these parameter during childhood growth and development is recommended. Moderate and balanced intake of VD, VA, and VE, as well as avoidance of excessive Pb and Cu exposure, may exert beneficial effects on the prevention and management of ADHD in children. In particular, given the strong and consistent association between VD deficiency and ADHD across both sexes and in multivariate analysis, routine assessment of VD status and appropriate supplementation should be considered as part of the clinical management of children with ADHD.

In addition, our study is limited by the lack of statistical date on potential confounding factors, including dietary intake, socioeconomic status, and sunlight exposure. The cross-sectional design precludes any causal inferences regarding the observed associations. Furthermore, the Hosmer-Lemeshow goodness-of-fit test indicated a less-than-optimal model fit (P < 0.001), suggesting that other important variables not included in our model may influence the relationship between micronutrients and ADHD. Future studies should take these confounding factors into account and may also adopt a longitudinal or interventional design to further explore the effects of nutritional supplements on ADHD symptoms. Additionally, the sample size in sex-stratified subgroups was relatively moderate, and larger studies are needed to confirm the sex-specific findings regarding Pb and VD.

## 5 Conclusion

In conclusion, serum deficiencies of VA, VD, and VE, as well as elevated levels of Pb and Cu, are associated with ADHD in children. VD deficiency shows the strongest and most consistent negative association with ADHD across both sexes and in multivariate analysis, while elevated Pb levels are associated with ADHD only in boys. The high prevalence of VK_1_ deficiency (25.8%) in the ADHD group represents a novel finding. Due to the cross-sectional design, causal inference cannot be made. Future longitudinal or interventional studies are needed to confirm these associations and evaluate the potential benefits of nutritional supplementation.

## Data Availability

The raw data supporting the conclusions of this article will be made available by the authors, without undue reservation.
