# Correction: Serum vitamins and microelements levels and their association with children with attention deficit hyperactivity disorder (ADHD)

**DOI:** 10.3389/fmolb.2026.2008863

**Published:** 2026-10-08

**Authors:** Tianzhu Chen, Jiaxiong Yao, Yang Su, Jingya Li, Panli Tan

**Affiliations:** 1 The First Affiliated Hospital of Zhejiang Chinese Medical University (Zhejiang Provincial Hospital of Chinese Medicine), Hangzhou, China; 2 Pharmacy Department, Preparation Center, Nanchang Hongdu Hospital of TCM, Nanchang, China; 3 China Jiliang University, Hangzhou, China

**Keywords:** ADHD, children, microelement, nervous disease, vitamin D

Author Yang Su was erroneously omitted as corresponding author. The correct corresponding authors are Yang Su, Jingya Li, Panli Tan.

The original version of this article has been updated.

