# Supplementary material for: Serum vitamins and microelements levels and their association with children with attention deficit hyperactivity disorder (ADHD)
Source: Front Mol Biosci. 2026 Aug 12;13:1786113. doi: 10.3389/fmolb.2026.1786113 (PMC13506877; doi:10.3389/fmolb.2026.1786113)
Supplement: Supplementary file 1 [file Table1.docx]

**Table 1 The Therapeutic range for serum vitamins levels.**

| **Variable** | **Therapeutic range** | **Detection limits(ng/mL)** |
| --- | --- | --- |
| Serum VA | Deficiency: ＜0.20μg/mL;  Marginal deficiency: 0.20-0.30 μg/mL;  Normal: 0.30-0.70μg/mL | 5-100000 |
| Serum VD_2_ | / | 3-1734.2 |
| Serum VD_3_ | / | 3-9961 |
| Serum VD | Seriously deficiency: ≤5.00ng/mL;  Deficiency: 5.01-15.00 ng/mL;  Insufficiency: 15.01-20.00 ng/mL;  Normal: 20.01-100.00 ng/mL;  Excess: ＞100.00 ng/mL | / |
| Serum VE | Normal: 3.80-18.40μg/mL | 150-199480 |
| Serum VK1 | Normal: 0.20-2.50 ng/mL | 0.15-208 |

**Table 2 The reference standards for serum microelements levels.**

| **Variable** | **Reference intervals** | **Detection limits(ng/mL)** |
| --- | --- | --- |
| Serum Cu | Normal: 9.42-27.06μmol/L | 4.945-20000 |
| Serum Ca | Normal: 1.35-2.25 mmol/L | 36.827-20000 |
| Serum Fe | Normal: 6.50-11.29 mmol/L | 46.553-200000 |
| Serum Mg | Normal: 1.35-2.07 mmol/L | 4.021-20000 |
| Serum Zn | Normal: 46.27-128.39μmol/L | 8.560-20000 |
| Serum Pb | Normal: 0.00-100.00μg/L | 0.082-200 |

**Table 3 Serum levels of vitamins and microelements in the HC group and ADHD group.**

| **Variable** | **HC** | **ADHD** | **χ^2^/t** | ***P*** | Cohen‘s d (95%CI) |
| --- | --- | --- | --- | --- | --- |
| Number of participant | 133 | 128 |  |  |  |
| Sex |  |  | 3.189 | 0.074 |  |
| Male | 57 (42.86%) | 69 (53.91%) |  |  |  |
| Female | 76 (57.14%) | 59(46.09%) |  |  |  |
| Age (years) | 9.42 ± 2.4 | 9.38 ± 2.5 | -0.153 | 0.878 | -0.02(-0.26,0.22) |
| Serum VA (μg/mL) | 0.43 ± 0.10 | 0.36 ± 0.11 | -5.1 | <0.0001**** | -0.63(-0.88,-0.38) |
| Serum VD_2_ (ng/mL) | 1.10 ± 2.14 | 0.92 ± 1.33 | -0.8 | 0.424 | -0.1(-0.34,0.14) |
| Serum VD_3_ (ng/mL) | 24.40 ± 5.43 | 16.56 ± 6.18 | -10.905 | <0.0001**** | -1.35(-1.62,-1.08) |
| Serum VE (μg/mL) | 10.10 ± 2.47 | 9.48 ± 2.54 | -1.984 | 0.048* | -0.25(-0.49,0) |
| Serum VD (ng/mL) | 25.50 ± 5.61 | 17.48 ± 6.41 | -10.766 | <0.0001**** | -1.33(-1.6,-1.06) |
| Serum VK_1_ (ng/mL) | 0.56 ± 0.42 | 0.50 ± 0.47 | -1.164 | 0.245 | -0.14(-0.39,0.1) |
| Serum Pb (μg/L) | 14.19 ± 6.50 | 19.15 ± 10.55 | 4.548 | <0.0001**** | 0.56(0.32,0.81) |
| Serum Zn (μmol/L) | 75.93 ± 11.25 | 76.44 ± 12.36 | 0.346 | 0.73 | 0.04(-0.2,0.29) |
| Serum Cu (μmol/L) | 14.60 ± 2.66 | 15.33 ± 2.9 | 2.126 | 0.034* | 0.26(0.02,0.51) |
| Serum Fe (mmol/L) | 8.12 ± 0.78 | 8.25 ± 0.77 | 1.351 | 0.178 | 0.17(-0.08,0.41) |
| Serum Ca (mmol/L) | 1.63 ± 0.18 | 1.64 ± 0.16 | 0.495 | 0.621 | 0.06(-0.18,0.3) |
| Serum Mg (mmol/L) | 1.72 ± 0.16 | 1.72 ± 0.17 | -0.027 | 0.978 | 0(-0.25,0.24) |
| ^*^:*P*＜0.05, ^**^:*P*＜0.01, ^***^:*P*＜0.001 ,^****^:*P*＜0.0001 compared with the HC group, n(%), median. | | | | | |

**Table 4 Serum levels of vitamins and microelements in the HC group and ADHD group(boys).**

| **Variable** | **HC(n=57)** | **ADHD(n=69)** | **χ^2^/t** | ***P*** | Cohen‘s d (95%CI) |
| --- | --- | --- | --- | --- | --- |
| Age (years) | 10.04 ± 2.88 | 9.94 ± 2.74 | 0.185 | 0.853 | 0.04 (-0.31, 0.39) |
| Serum VA (μg/mL) | 0.43 ± 0.11 | 0.37 ± 0.12 | 3.16 | 0.002** | 0.57 (0.21, 0.93) |
| Serum VD_2_ (ng/mL) | 1.12 ± 2.43 | 1.04 ± 1.57 | 0.219 | 0.827 | 0.04 (-0.31, 0.39) |
| Serum VD_3_ (ng/mL) | 24.91 ± 6.59 | 16.54 ± 6.32 | 7.254 | <0.001*** | 1.30 (0.90, 1.70) |
| Serum VE (μg/mL) | 9.65 ± 2.33 | 9.29 ± 2.25 | 0.873 | 0.384 | 0.16 (-0.19, 0.51) |
| Serum VD (ng/mL) | 26.03 ± 6.31 | 17.58 ± 6.57 | 7.308 | <0.001*** | 1.31 (0.91, 1.71) |
| Serum VK_1_ (ng/mL) | 0.60 ± 0.55 | 0.49 ± 0.54 | 1.124 | 0.263 | 0.20 (-0.15, 0.55) |
| Serum Pb (μg/L) | 14.19 ± 6.44 | 21.96 ± 11.02 | -4.698 | <0.001*** | -0.84 (-1.21, -0.47) |
| Serum Zn (μmol/L) | 77.15 ± 10.67 | 78.89 ± 12.07 | -0.852 | 0.396 | -0.15 (-0.50, 0.20) |
| Serum Cu (μmol/L) | 15.21 ± 2.99 | 15.66 ± 2.92 | -0.86 | 0.392 | -0.15 (-0.50, 0.20) |
| Serum Fe (mmol/L) | 8.31 ± 0.83 | 8.47 ± 0.79 | -1.147 | 0.254 | -0.21 (-0.56, 0.14) |
| Serum Ca (mmol/L) | 1.61 ± 0.21 | 1.62 ± 0.16 | -0.269 | 0.789 | -0.05 (-0.40, 0.30) |
| Serum Mg (mmol/L) | 1.73 ± 0.15 | 1.71 ± 0.17 | 0.805 | 0.422 | 0.14 (-0.21, 0.49) |
| ^*^:*P*＜0.05, ^**^:*P*＜0.01, ^***^:*P*＜0.001 compared with the HC group, n(%), median. | | | | | |

**Table 5 Serum levels of vitamins and microelements in the HC group and ADHD group(girls).**

| **Variable** | **HC(n=77)** | **ADHD(n=58)** | **χ^2^/t** | ***P*** | Cohen‘s d (95%CI) |
| --- | --- | --- | --- | --- | --- |
| Age (years) | 8.94 ± 1.85 | 8.74 ± 1.90 | 0.597 | 0.552 | 0.11 (-0.23, 0.45) |
| Serum VA (μg/mL) | 0.42 ± 0.09 | 0.36 ± 0.11 | 3.929 | <0.001*** | 0.70 (0.34, 1.06) |
| Serum VD_2_ (ng/mL) | 1.08 ± 1.89 | 0.78 ± 0.98 | 1.103 | 0.272 | 0.20 (-0.14, 0.54) |
| Serum VD_3_ (ng/mL) | 23.92 ± 4.45 | 16.60 ± 6.11 | 8.06 | <0.001*** | 1.44 (1.04, 1.84) |
| Serum VE (μg/mL) | 10.46 ± 2.53 | 9.66 ± 2.84 | 1.722 | 0.087 | 0.31 (-0.03, 0.65) |
| Serum VD (ng/mL) | 24.99 ± 5.09 | 17.37 ± 6.33 | 7.758 | <0.001*** | 1.38 (0.99, 1.78) |
| Serum VK_1_ (ng/mL) | 0.53 ± 0.28 | 0.50 ± 0.40 | 0.46 | 0.647 | 0.08 (-0.26, 0.42) |
| Serum Pb (μg/L) | 14.30 ± 6.61 | 15.76 ± 9.03 | -1.085 | 0.28 | -0.19 (-0.53, 0.15) |
| Serum Zn (μmol/L) | 75.18 ± 11.66 | 73.34 ± 12.15 | 0.894 | 0.373 | 0.16 (-0.18, 0.50) |
| Serum Cu (μmol/L) | 14.73 ± 2.80 | 14.71 ± 2.25 | 0.089 | 0.93 | 0.01 (-0.33, 0.35) |
| Serum Fe (mmol/L) | 7.98 ± 0.71 | 7.97 ± 0.67 | 0.117 | 0.908 | 0.02 (-0.32, 0.36) |
| Serum Ca (mmol/L) | 1.64 ± 0.17 | 1.66 ± 0.16 | -0.613 | 0.541 | -0.11 (-0.45, 0.23) |
| Serum Mg (mmol/L) | 1.71 ± 0.16 | 1.73 ± 0.17 | -0.739 | 0.461 | -0.13 (-0.47, 0.21) |
| ^*^:*P*＜0.05, ^**^:*P*＜0.01, ^***^:*P*＜0.001 compared with the HC group, n(%), median. | | | | | |

**Table 6 The categorical distributions of serum vitamins**

| Groups | | Deficiency | Normal | Excess |
| --- | --- | --- | --- | --- |
| Vitamin A | HC | 9（6.8%） | 124（93.2%） | 0 |
|  | ADHD | 44（34.4%） | 84（65.6%） | 0 |
| Vitamin E | HC | 0 | 133（100%） | 0 |
|  | ADHD | 1（0.8%） | 127（99.2%） | 0 |
| Vitamin D | HC | 0 | 133（100%） | 0 |
|  | ADHD | 95（74.2%） | 33（25.8%） | 0 |
| Vitamin K_1_ | HC | 0 | 133（100%） | 0 |
|  | ADHD | 33（25.8%） | 94（73.4%） | 1（0.8%） |
| Zn | HC | 1（0.8%） | 132（99.2%） | 0 |
|  | ADHD | 1（0.8%） | 127（99.2%） | 0 |
| Cu | HC | 0 | 133（100%） | 0 |
|  | ADHD | 0 | 127（99.2%） | 1（0.8%） |
| Fe | HC | 2（1.5%） | 131（98.5%%） | 0 |
|  | ADHD | 1（0.8%） | 127（99.2%） | 0 |
| Ca | HC | 2（1.5%） | 131（98.5%%） | 0 |
|  | ADHD | 0 | 128（100%） | 0 |
| Mg | HC | 0 | 133（100%） | 0 |
|  | ADHD | 1（0.8%） | 127（99.2%） | 0 |

**Table 7 Difference of various indicators between ADHD patients of different gender.**

| Groups | Male 69（53.9%） | Female 59（46.1%） | *P* value | F value |
| --- | --- | --- | --- | --- |
| VA (μg/mL) | 0.37±0.12 | 0.35±0.11 | 0.441 | 1.297 |
| VD_2_ (ng/mL) | 1.04±1.57 | 0.78±0.97 | 0.258 | 2.627 |
| VD_3_ (ng/mL) | 16.54±6.32 | 16.58±6.06 | 0.970 | 1.086 |
| VE (μg/mL) | 9.29±2.25 | 9.70±2.84 | 0.363 | 1.591 |
| VD (nmol/L) | 17.58±6.57 | 17.36±6.28 | 0.844 | 1.095 |
| VK_1_ (ng/mL) | 0.49±0.54 | 0.50±0.39 | 0.931 | 1.887 |
| Pb (μg/L) | 21.96±11.02 | 15.86±8.99 | <0.001*** | 1.504 |
| Zn (μmol/L) | 78.89±12.07 | 73.57±12.17 | 0.015* | 1.018 |
| Cu (μmol/L) | 15.66±2.92 | 14.95±2.86 | 0.165 | 1.039 |
| Fe (mmol/L) | 8.47±0.79 | 7.98±0.67 | <0.001*** | 1.404 |
| Ca (mmol/L) | 1.62±0.16 | 1.66±0.16 | 0.168 | 1.022 |
| Mg (mmol/L) | 1.71±0.17 | 1.73±0.17 | 0.594 | 1.073 |
| **P*<0.05; ****P*<0.001 | | | | |

**Table 8 Binary Logistic Regression Analysis of the Associations between Vitamins (A, E, D), Microelements (Cu, Pb) and ADHD.**

| Predictor | B (SE) | Wald χ² | P-value | Adjusted OR | | 95% CI | |
| --- | --- | --- | --- | --- | --- | --- | --- |
| **Demographic Factors** | |  |  |  |  | |  |
| Age (per 1-year increase) | -0.13(0.08) | 2.585 | 0.108 | 0.88 | (0.75,1.03) | |  |
| Sex (Ref: male) | 0.4(0.35) | 1.336 | 0.248 | 1.498 | (0.76,2.97) | |  |
| **Fat-Soluble Vitamins** | |  |  |  |  | |  |
| Vitamin A | -2.05(1.63) | 1.587 | 0.208 | 0.129 | (0.01,3.12) | |  |
| Vitamin D | -0.26(0.04) | 45.047 | <0.001**** | 0.774 | (0.72,0.83) | |  |
| Vitamin E | -0.02(0.07) | 0.053 | 0.818 | 0.985 | (0.86,1.12) | |  |
| **Essential Metals & Minerals** | | |  |  |  | |  |
| Lead (Pb) | 0.05(0.03) | 3.751 | 0.053 | 1.049 | (1,1.1) | |  |
| Copper (Cu) | 0.04(0.07) | 0.369 | 0.544 | 1.04 | (0.92,1.18) | |  |
| **Model Summary:** N = 261. The full model was statistically significant: χ²(14) = 121.94, p< 0.001. Hosmer-Lemeshow goodness-of-fit test: χ²(8) = 55.58, p< 0.001. Nagelkerke R² =0.5. | | | | | | |  |
